# Supplementary figures and images for: The leucine-NH4+ uptake regulator Any1 limits growth as part of a general amino acid control response to loss of La protein by fission yeast
Source: PLoS One. 2021 Jun 21;16(6):e0253494. doi: 10.1371/journal.pone.0253494 (PMC8216550; doi:10.1371/journal.pone.0253494)

# Raw images for figure 2

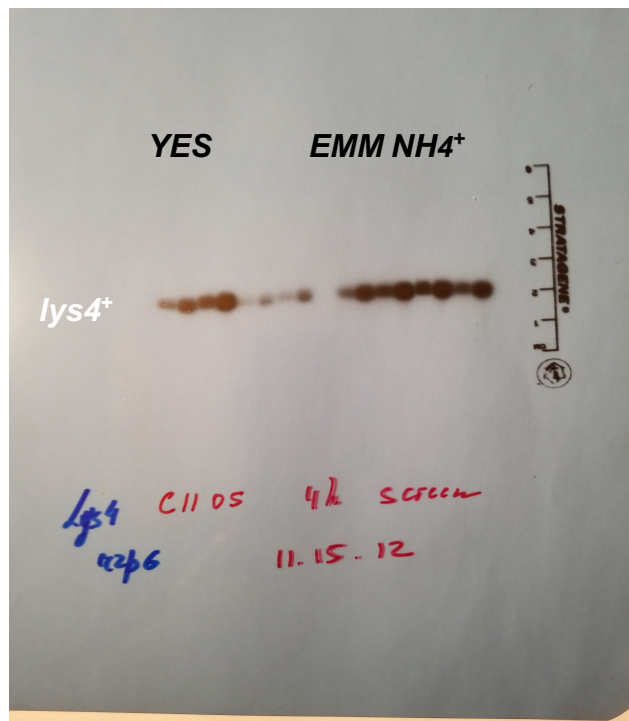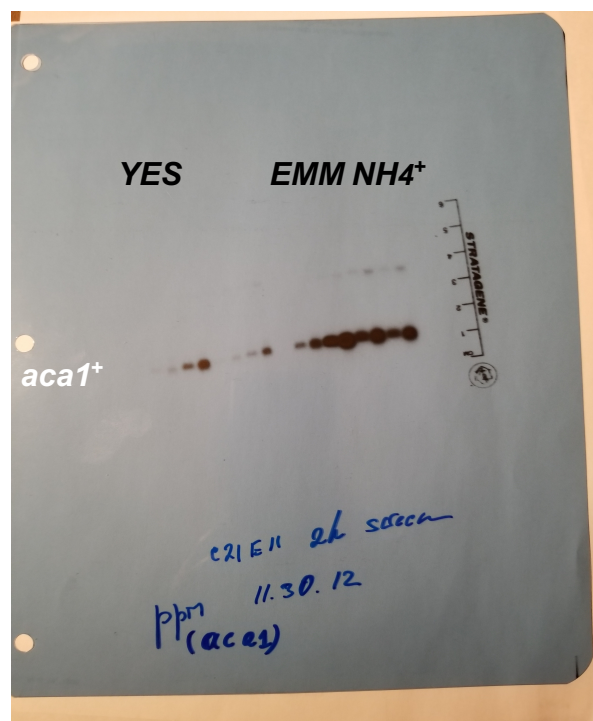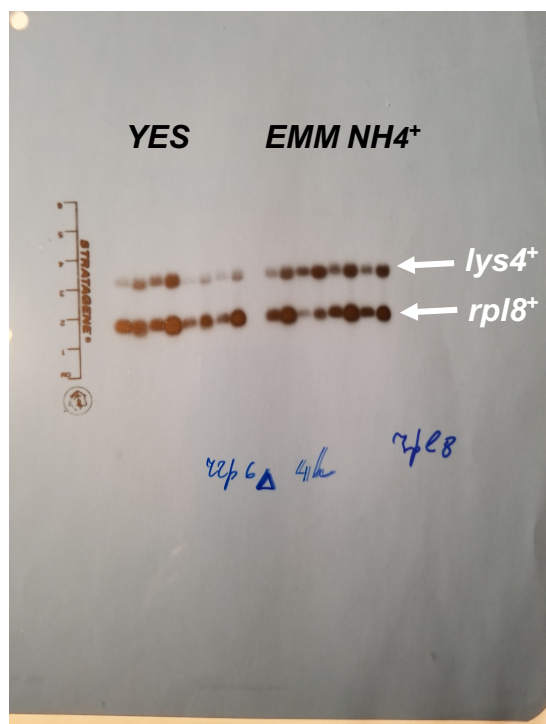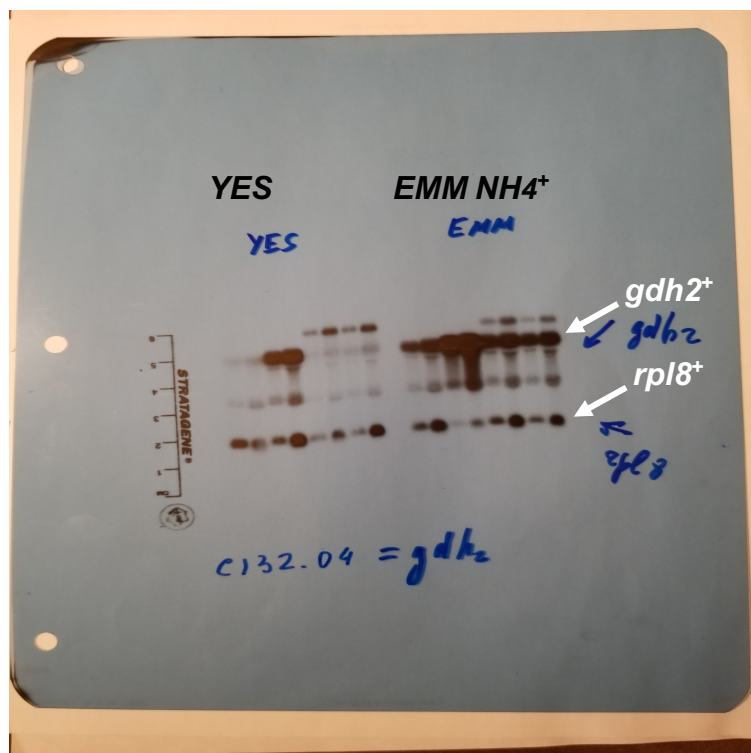

# Raw images for figure 3

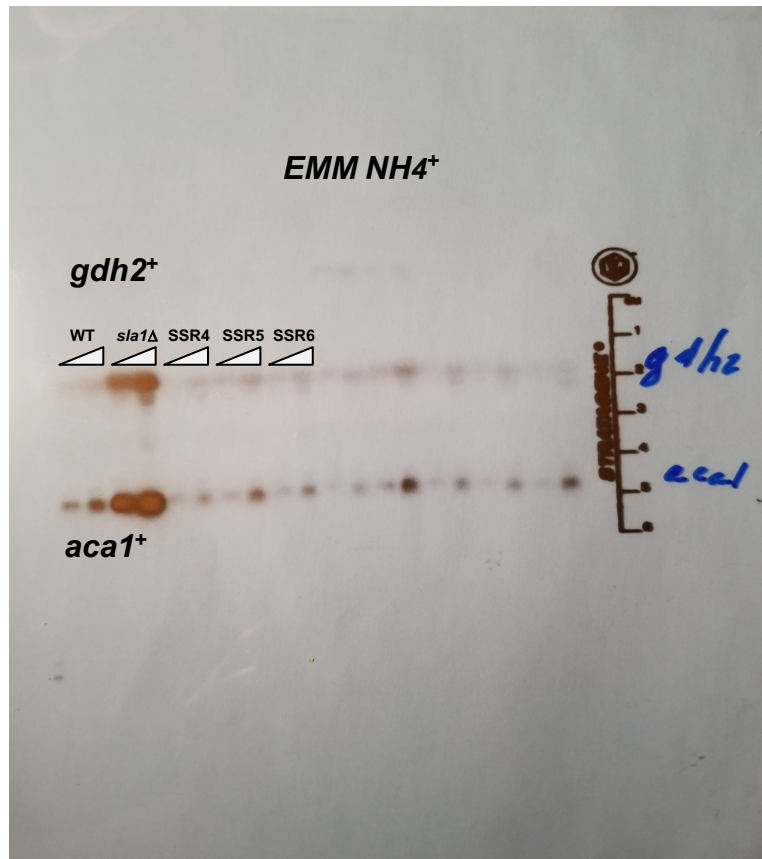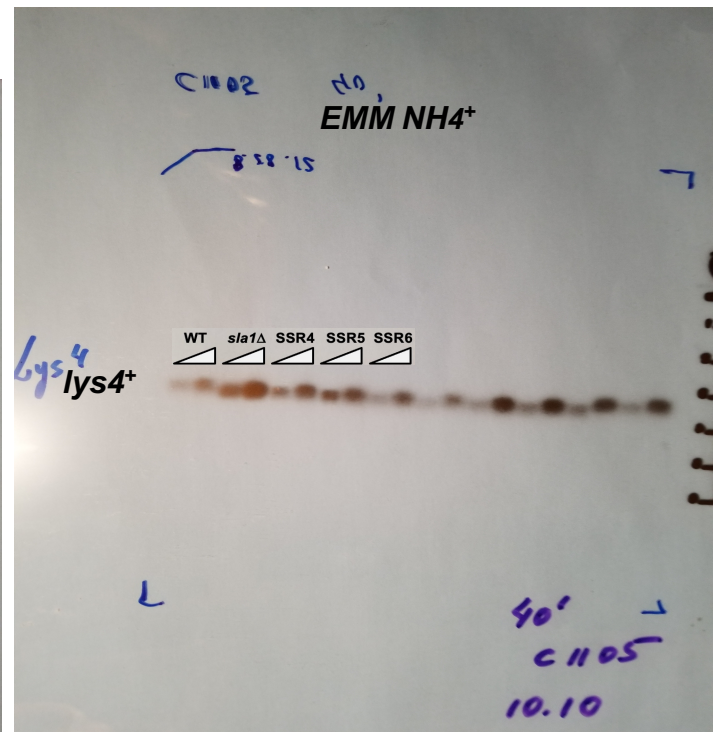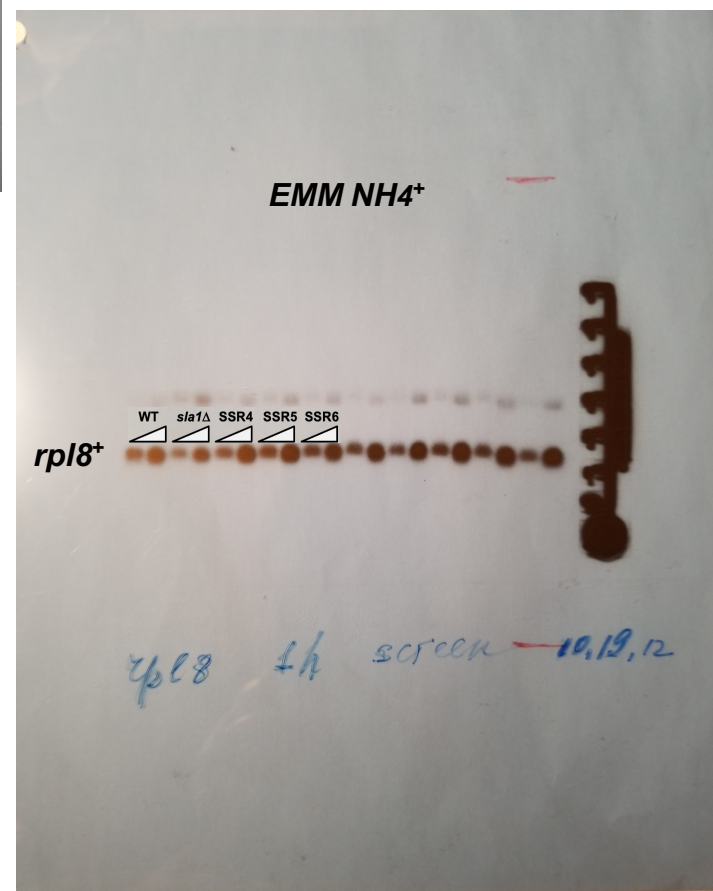

# Raw images for figure 5

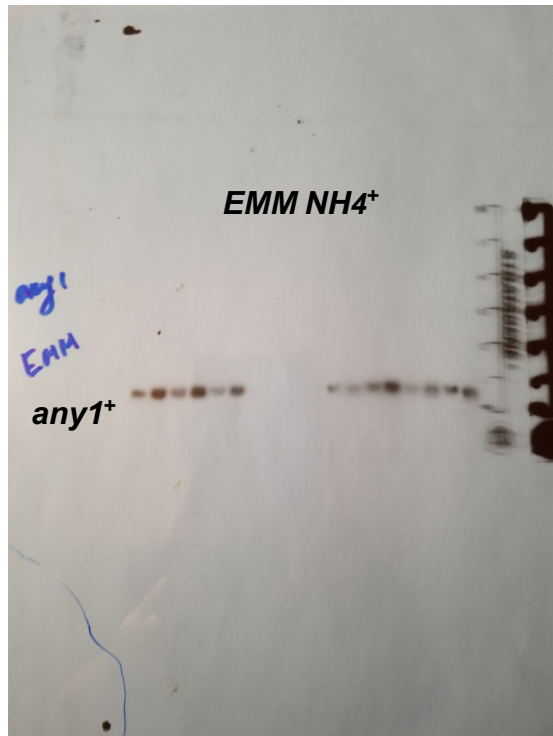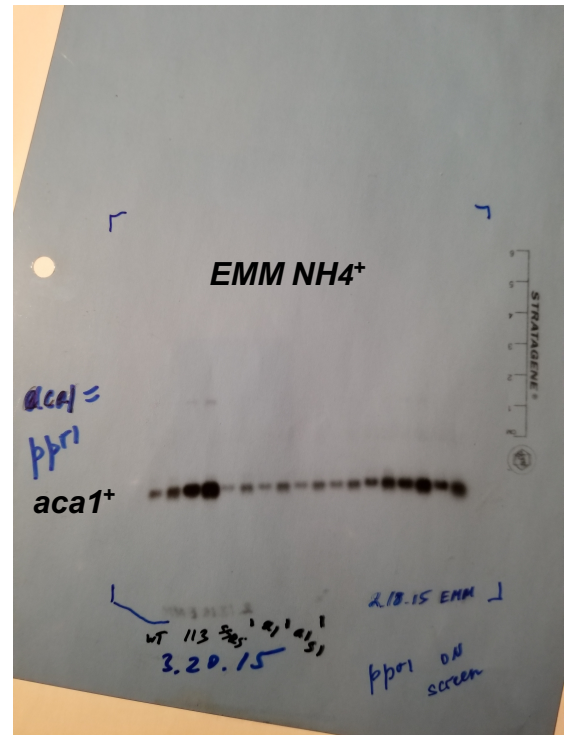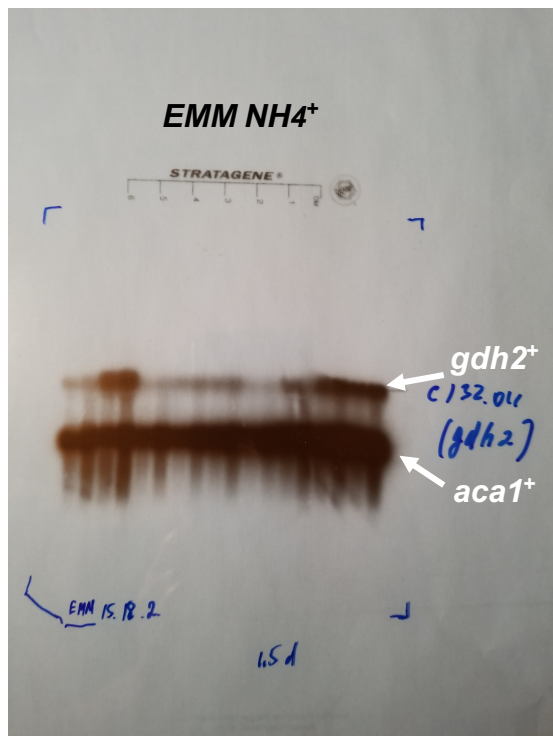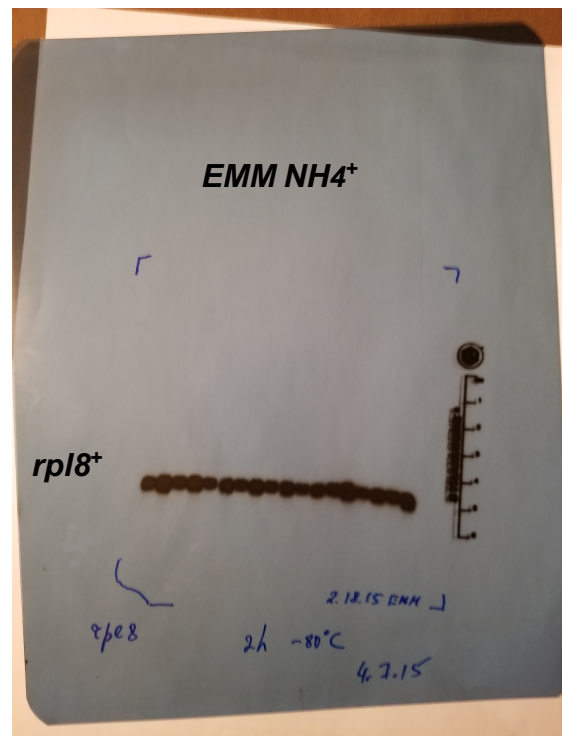

Supplement: S1 Raw image — (PDF) [file pone.0253494.s008.pdf]
