# Supplementary material for: The leucine-NH4+ uptake regulator Any1 limits growth as part of a general amino acid control response to loss of La protein by fission yeast
Source: PLoS One. 2021 Jun 21;16(6):e0253494. doi: 10.1371/journal.pone.0253494 (PMC8216550; doi:10.1371/journal.pone.0253494)
Supplement: Attachment — Submitted filename: Response to Reviewers PLoS One Any1R1.pdf [file pone.0253494.s009.pdf]

Cherkosova et al., Maraia, PONE-D-21-06328.
The leucine-NH4+ uptake regulator Any1 limits growth as part of a general amino acid stress response to loss of La protein by fission yeast

Point-by-point responses

Reviewer #1: In the manuscript submitted by Cherkasova et al, the authors provide the evidence that the sla1+ gene of S. pombe, which encodes La protein, mediates general amino acid control (GAAC) response concomitant with nuclear surveillance mechanisms. First, transcriptome analysis reveals that genes upregulated in sla1D cells exhibit highly significant overlap with GAAC genes. Second, GAAC gene upregulation is suppressed by rrp6 deletion. Third, the authors isolated sla1D spontaneous revertant (SSR), which suppresses slow growth phenotype in NH4 + -media, and identified an F32V mutation of any1+ gene in the SSR mutants by whole genome sequencing. Furthermore, 3H-leucine uptake of SSR- any1-F32V cells in NH4 + -media is more robust than by sla1D cells.
•Author response: We appreciate the careful reading and supportive conclusions.

The manuscript presents noble findings regarding a function of sla1+ gene, and is suitable for PLOS ONE. However, several important information is missing as described below, therefore, I cannot support publication of the manuscript in its present form.
•Author response: We appreciate the assessment. Information referred to as missing has been amended in the revised manuscript (MS), as described below.

The mention of "missing information" by the Reviewer appears to refer to strengthening data that is already reported in the manuscript by adding more of it to bolster its statistical significance. As will be described below for each specific comment, a figure, manuscript result section and/or figure legend has been revised by the addition of relevent data and/or appropriate text description.

A brief summary of the additions/edits in response to Reviewers' comments:

Fig 2: i) Addition of EtBr-stained gel panel to help interpret the loading control for *rpl8*$^+$ mRNA.
ii) Reconfiguration of bars in graph to make more clear and distinct.
iii) Addition of panel C) with results of ***p*-values** representing statistical analysis of quantitative data.

Fig 3: i) Addition of **quantification** values for *gdh2*$^+$ and *aca1*$^+$ mRNAs under the lanes.
ii) Addition of the EtBr-stained gel panel to help interpret the loading control for *rpl8*$^+$ mRNA.

Fig 5: i) Additional **quantification data with SDs** for *gdh2*$^+$ and *aca1*$^+$ mRNAs under the lanes.

Fig 6: i) Addition of **R^2 values** to represent fit of the lines that represent the (slope) rates of leucine uptake.
ii) Addition of panel B) with results of ***p*-values** representing statistical analysis of leucine uptake.

Specific comments:

1. The authors showed that sla1D cells exhibited increased levels of gdh2+ and aca1+ mRNAs in EMM-NH4+ -media, which has already been shown by the authors' previous study (in ref [50]). And then, they showed that additional rrp6D (Fig 2) and any1D (Fig 5) mutations reduced the upregulation. The levels of the increase of gdh2+ mRNA in sla1D cells is not comparable between the experiments in Fig 2 and Fig 5; it was detected at approx. x8 (Fig 2B, right) and x3.6 (Fig 5, lanes 3-4), respectively. It is also the case for aca1+ mRNA; it was detected at approx. x6 (Fig 2B, right) and x4.5 (Fig 5, lanes 3-4). Is there any difference in experimental conditions between these experiments? Is this just within the experimental variations? The authors need to perform these experiments at least three times and present the results with standard deviations and p-values.
•Author response: The reviewer's main concern here is variability of the increase in *gdh2*$^+$ and *aca1*$^+$ mRNAs in *sla1*Δ relative to WT cells in EMM-NH_4^+ media in different experiments as monitored by northern blotting, namely Fig 2B right (EMM-NH_4^+) and Fig 5 (lanes 3-4, EMM-NH_4^+). We had been aware of this issue. Here we note our oversight, that in addition to the two northern blots referred to by the Reviewer, a third, Fig 3E, which we realize had not been specified as from cells grown in EMM-NH_4^+ can be added which we noted in the revised figure legend was also grown in EMM-NH_4^+. We therefore also added quantification values below the lanes of the two relevent panels of Fig 3E (also noted in the legend) and also as suggested by this Reviewer, we strengthened the data for Fig 5 by incorporating additional quantification measurements from independent experiments. The levels for *gdh2*$^+$ and *aca1*$^+$ that are shown in revised Fig 3E are intermediate between those for Figs 2 & revised Fig 5. We can now state for Figures 2, 3 & 5, the quantification values reported were derived from measurements of two separate RNA sample loadings per experiment (technical duplicates), and for Fig 5 duplicate experiments, hve been strengthened. We thus

have four northern blot quantitative comparisons of *sla1*Δ and WT cells grown in EMM-NH_4^+, each from at least technical duplicates. These show that, *gdh2*$^+$ was 3.1X-to-8.2X induced and *aca1*$^+$ was 3.7X-to-6.2X induced in four independent experiments. There is more variability in the *gdh2*$^+$ data. However, there is no doubt that *gdh2*$^+$ and *aca1*$^+$ are more than 2-fold induced in *sla1*Δ cells relative to WT cells in EMM-NH_4^+ further confirming the gene-array results for GAAC genes which are more collectively represented in Figure 1 and with stronger statistical significance. However, this is not the issue. The Reviewer noted that these mRNAs have already been characterized as upregulated EMM-NH4+ -media in his/her reference to our previous study (in ref [50]) which was by gene array technology which is recognized as superior to northern blotting for such quantification. We contend here that assignment of standard deviation and *p*-values to the northern blot data for the differences in *gdh2*$^+$ and *aca1*$^+$ among different experiments is not worthy of such exercise here, and would be relevent only as a minor conclusion of Figure 2. The more important and major conclusion of Fig 2 is suppression of GAAC induction by *rrp6*-deletion due to the biological implications of a signaling component of nuclear surveillance in the first species other than *S. cerevisiae*. This we have strengthened the statistical significance of the quantitative data (below).

I appreciate the general and honorable concern of Reviewer #1 for variability in data, and we have strengthened the statistical significance of the data throughout as described below. We added a paragraph that better describes the relative quantitative aspects of the northern blot and effects of *rrp6*-deletion in Fig 2A,B and variability which appears under the figure 2 legend (p.12). We added two sentences to the Fig 2 legend, that "duplicates" contributed to quantitation, and that addition of the EtBr gel panel will help interpretation of the loading control *rpl8*$^+$ mRNA, a topic of comment 2 below. In addition we added a new panel (C) to Fig 2 that contains *p*-values for the quantitative data on *gdh2*$^+$ and *aca1*$^+$ mRNAs.

We thank the Reviewer for asking if differences observed from one experiment to another is "just within the experimental variations?" Our answer is that variability indeed appears to be significant and that *sla1*Δ cells may be unusually sensitive to dynamic changes that occur in nutrient levels during growth which may account for unwanted experimental variations. The regulatory mechanisms involved are known to be highly sensitive to nutrients in the media. The mRNAs examined in the figures discussed above encode enzymes involved in nutrient metabolism. Gdh2 (NAD^+-dependent glutamate dehydrogenase) is key in nitrogen metabolism, central to ammonia and linked to anabolism. The *S. cerevisiae* homolog, *GDH2* for which more is known than for *gdh2*$^+$, is transcriptionally controlled by six different sequence elements upstream of the promoter and sensitive to multiple nutrients. As reported in our manuscript, *zfs1*$^+$ was the target of mutation in SSR6 and that it has more promoters than the great majority of *S. pombe* genes, it undergoes nitrogen-responsive transcription, and that *zfs1*$^+$ controls the G1 cyclin *puc1*$^+$ in response to nitrogen depletion (pp 15-16 and refs therein). Also as noted, plates in Fig 3 show that as SSR mutants grow, *sla1*Δ satellite colonies emerge and grow surrounding them most likley because the NH_4^+ is dynamically depleted during growth of the SSR as part of anabolic processes. This surprising data together with the other observations provide evidence that nutrient variability that occurs dynamically during cell growth is sufficient to dramatically alter *sla1*Δ cell growth due to consequent gene regulation. Our unpublished data show that over-expression of Gdh2 promotes growth specifically in *sla1*Δ cells, in EMM-NH_4^+. These collective observations comprise the basis for our answer yes, observed experimental variations may be because *sla1*Δ cells are unusually sensitive to dynamic changes in nutrient levels during cell growth. In any case, we emphasize that in the three independent experiments the *gdh2*$^+$ and *aca1*$^+$ variability between *sla1*Δ and WT cells was limited to the range above 2-fold (including Fig 5), again re-confirming the gene array data that we previously reported.

2. Expression levels of rpl8+ mRNA in sla1D cells grown in EMM-NH4+ -media is drastically reduced in the experiment in Fig 2A (lanes 11 and 12), but not in Fig 3E (lanes 3 and 4) and Fig 5 (lanes 3 and 4). If the authors perform these experiments under the same conditions, the difference should not be appeared.

•Author response: I thank the Reviewer for pointing out the apparent visual differences in *rpl8*$^+$ levels. We agree with the point that performing experiments under the same conditions should produce no differences, and also note that as noted in the paragraph above *sla1*Δ appears to be unusually sensitive to variabilities. Nonetheless, a problem was that the *rpl8*$^+$ mRNA was used as a loading control for the *gdh2*$^+$ and *aca1*$^+$ mRNAs on the northern blots but the EtBr-stained gel photos showing general RNA loading levels was not provided for Figs 2 and 3. I revised the figures by adding the EtBr-stained gel photos under the northern blot panels of Fig 2 and Fig 3. This reveals for Fig 2 that the *sla1*Δ cells in lane 11 which has the reduced amount of *rpl8*$^+$ mRNA noted by the Reviewer also has a low level of RNA loaded as seen by comparison of EtBr 26S rRNA levels. The EtBr gel added to Fig 3 also helps with such

assessment. We agree that the *rpl8⁺* mRNA levels are down in *sla1*Δ cells. This might not be unexpected in cells disrupted for TORC1 signaling which otherwise positively controls production of individual ribosomal proteins as well as other aspects of ribosome biosynthesis. Investigating this is beyond the scope of this study. Fig 5 had an EtBr-stained gel; comparison of the levels of 26S and 18S rRNA in lanes 3 & 4 vs 1 & 2 relative to the *rpl8*$^+$ mRNA levels indicate that as the Reviewer's comments intended, this experiment reveals more variability than the other two.

In response to this Reviewer's comment 5 below, we revised Fig 5 by adding more data based on additional experiments that increased the statistical strength of the *any1*-allele data reported in the top two panels. The revised data were derived from a different northern blot also normalized to *rpl8*$^+$ mRNA levels (below), it provided more data which contributes to the confidence in the trend in the data and statistical significance.

3. It seems that the authors isolate five SSRs (SSR1-5), whose growth phenotype was shown in Fig 3C. SSR1 also shows robust suppression phenotype in addition to SSR4, SSR5 and SSR6. Why do they abandon further analysis for SSR1 including whole genome sequencing? The authors should describe the precise clone number of isolated SSR and the reason why they pick SSR4, SSR5 and SSR6 in the manuscript.

•Author response: We thank the Reviewer for pointing out that our description lacked clarity. This comment and comment 4 below refer serial dilution spot assays of a variety of candidate colony subclones as well as sattelite colony clones that represented an intermediate screening stage of our selection process. The previous Fig 3C (removed in the revised MS) had designated such colonies as SSR-2b, SSR-3b, SSR-4b, ssc-1s that were confusing and not referred to later in the MS. Therefore, we believe that it was best to remove panel C. We edited the text accordingly which is now more succint and relevent in the revised manuscript. With regard to precise clone number of isolated SSRs, the official designation names in our records and of the frozen stocks in our lab that were used for whole genome sequencing are SSR4, SSR5 and SSR6. The new more descriptive edited paragraph on p.13 is copied here (note that Fig 3C in the last sentence refers to 3D in the original MS version):

> **Isolation of *sla1*Δ spontaneous revertants (SSR) of slow growth in NH_4^+ media.** *sla1*Δ cells plated on EMM-NH_4^+ led to the appearance of very rare relatively fast growing colonies that were reproducibly surrounded by smaller sattelite colonies (Fig 3A). After plating 125,000 cells, a rare colony arose and satellite colonies appeared thereafter as in Fig 3B. The fast growth colonies isolated after a single plating were named *sla1*Δ spontaneous revertants (SSRs) followed by a number. These were streaked and single colony derivatives of each were screened by comparing their growth to WT, *sla1Δ* and small sattelite colonies, by serial dilution spotting on YES, EMM-NH_4^+ and EMM-Pro. Additional analyses are shown in Fig 3C.

4. SSRs were indicated like "SSR-4b" but not "SSR4" in Fig 3C. What is the difference between SSRX and SSR-Xb? The authors should describe the difference in the figure legend or main text.

•Author response: See above, response to comment 3.

5. In Fig 5, the authors claimed that any1-F32V led to lower aca1+ mRNA levels than any1+ in the sla1D background (compare lanes 15-16 and 17-18, x3.6 and x2.1, respectively). However, the aca1+ mRNA levels of sla1D any1D double mutant that is integrated sla1+ is also lower than that of sla1D (compare lanes 3-4 and 15-16, x4.5 and 3.6, respectively), which should be comparable, considering the authors claim as described in lines 405-407. The result is not supportive. To confirm the claim, the authors need to present the results with standard deviations and p-values as described above and reevaluate the result.

•Author response: We thank the Reviewer for these insightful comments which when addressed are helpful toward understanding the relationship between restoration of *aca1*$^+$ mRNA levels and growth in EMM-NH_4^+ conferred by *any1-F32V* that distinguish this allele from the *any1*$^+$ allele after these were integrated into the double mutant *sla1*Δ *any1*Δ. In addressing this here we first note that the *aca1*$^+$ and *gdh2*$^+$ mRNA levels in revised Fig 5 benefitted from statistical strengthening in response to this Reviewer's specific comment 1. The Reviewer noted a weakness about the data that was "not supportive." We note that this had not been properly acknowledged in our previous manuscript and that more thorough discussion of these data would be appropriate. We have significantly revised this section of the Results, including by addressing the issue referred to by the Reviewer. This section was expanded by lengthening the paragraph and adding two short paragraphs describing the data and 'reevaluating as suggested.Although the trend of the *aca1*$^+$ and *gdh2*$^+$ mRNA data (Fig 5) and the relationship of the *aca1*$^+$ mRNA levels to the growth of the strains in

EMM-NH_4^+ remain the same (Fig 4B), this part of the manuscript has been strengthened in part because of our considerations of response to the issues raised by the Reviewer. The edited paragraphs including revised Fig legend 5 are on pp.17-18.

> We next examined effects of integrating *any1*$^+$ and *any1-F32V* into the *any1*Δ strain and the double mutant *sla1*Δ *any1*Δ (Fig 5, lanes 11-18). Both alleles restored *any1*$^+$ mRNA levels to the *any1*Δ and *sla1*Δ *any1*Δ strains (*any1*$^+$ panel, compare lanes 7-10 with 11-18). The *any1-F32V* allele in lanes 17-18 failed to recapitulate *gdh2*$^+$ and *aca1*$^+$ levels comparable to that in SSR5 (lanes 5-6). However, examination of the blot and quantification suggested that *any1-F32V* suppressed *aca1*$^+$ expression more than it suppressed *gdh2*$^+$ (lanes 15-18). Thus although *any1-F32V* failed to recapitulate *gdh2*$^+$ and *aca1*$^+$ levels observed in SSR5, it appeared to control *aca1*$^+$ levels more than *gdh2*$^+$. More specifically, the *any1-F32V* allele led to lower *aca1*$^+$ levels than the *any1*$^+$ allele after integration *into sla1*Δ *any1*Δ, 5.0 +/-2.0 vs 2.75 +/-1.0 (lanes 15-16 vs. 17-18), whereas *gdh2*$^+$ levels were more similar at 5.4 +/-2.0 vs 5.4 +/-2.9 (lanes 15-16 vs. 17-18). Although these quantifications do not carry strong statistical significance, visual inspection of the northern blot internal controls provide additional evidence that the *any1-F32V* allele is more effective at suppressing *aca1*$^+$ levels than is the *any1*$^+$ allele. This is assessed by comparing relative band intensities in lanes 15-18 for *rpl8*$^+$, *any1*$^+$ and *aca1*$^+$ mRNAs in Fig 5. It reveals that while *rpl8*$^+$ and *any1*$^+$ show stronger band intensities in both lanes 15-16 as compared to both corresponding lanes 17-18, the band intensities for *aca1*$^+$ mRNA are reversed, with stronger signals in lanes 17-18 as compared to lanes 15-16.
>
> The data show that integration of neither *any1-F32V* nor *any1*$^+$ could recapitulate the suppression levels of *gdh2*$^+$ and *aca1*$^+$ observed in SSR5. The data suggested that *any1-F32V* was partially effective as its integration suppressed *aca1*$^+$ levels to greater extent than *any1*$^+$. Specifically, *aca1*$^+$ levels in the *any1-F32V* integrant was intermediate between *sla1*Δ and SSR5 (Fig 5). The data are consistent with the idea that re-integtation of *any1-F32V* and *any1*$^+$ into the *sla1*Δ*any1*Δ double mutant failed to re-establish all of the regulatory control regions of these genes.
>
> Are the observations regarding the apparent differential *aca1*$^+$ mRNA levels relevent to the growth of the strains in EMM-NH_4^+? Fig 4B shows that while *sla1*Δ and *sla1*Δ*any1*Δ+*any1*$^+$ show comparable poor growth, *sla1*Δ*any1*Δ+*any1-F32V* exhibits intermediate level growth, not as good as SSR5 but clearly better than *sla1*Δ *any1*Δ +*any1*$^+$. The data confirm that *any1-F32V* is a growth determinant in *sla1*Δ-SSR5 in EMM-NH_4^+ distinguished from *any1*$^+$, and this would appear to be somewhat correlated with effects on *aca1*$^+$ mRNA levels.

6. Point mutation sometimes effects on the stability of the protein. The authors need to confirm any1+ and any1-F32V protein levels, in addition to the mRNA expression levels by northern blotting shown in Fig 5.

•Author response: We agree that point mutation may alter protein stability. This may be relevent. However, although as noted by the Reviewer, we showed that *any1*$^+$ and *any1-F32V* produce mRNA at similar levels, the mechanism by which *any1-F32V* exerts its effect beyond this was not intended to be part of our manuscript. The last paragraph discusses issues related to stability of Any1-Pub1 interactions.

Our growth assays in Fig 4C show that *any1*$^+$ and *any1-F32V* are comparable at rescuing the slow growth phenotype of *sla1*Δ*any1*Δ in YES, whereas they markedly differ in EMM-NH_4^+. These and collective data are consistent with a qualitative effect of F32V. We believe that if Any1 protein instability is due to the F32V mutation, it may likley be regulatory in nature and differentially occurs in *sla1*Δ cells in EMM-NH_4^+. We believe that if the F32V mutation affects Any1 protein instability it may do so by influencing regulatory ubiqutinylation. We don't believe that we should be compelled to confirm Any1 protein levels because it is not a simple issue and the proper investigation is beyond the scope of this paper. The last sentence of the paper states: "The Any1-F32V allele may be useful in future studies that seek to understand mechanisms involved in such regulation."

7. The authors conclude that sla1D mutant exhibited decreased leucine uptake in EMM-NH4+ media compared to WT, in addition, any1D mutant, sla1D any1D double mutant, and SSR5 exhibited greater uptake than WT (lines 412-414). To confirm these differences, again, the authors need to present the results with standard deviations and p-values.

•Author response: We thank the Reviewer for the comments, which have led to an improved manuscript. I am happy to report that as expected of a series of near near straight lines each comprised of more than two points, statistical analysis applied to the data in Figure 6 are strongly supportive. Multiple regression/correlation analysis was used to

derive *p*-values which were added as a B panel to the Fig 6. In addition, trend lines were derived for the data points on the graph in Fig 6A and the R^2 values for each of these, which represent the slopes of the rates of leucine incorporation, were added.

Reviewer #2: The manuscript entitled "The leucine-NH4+ uptake regulator Any1 limits ..." by Cherkasova et al. first analyzed gene expression profiles of a sla1Δ mutant, lacking a tRNA processing factor La homologue in S. pombe, grown in three different N-source conditions and found that the mutant exhibits significant similarity to the wild-type cells under GAAC in transcriptome. In addition, the sla1Δ cells in NH4+ media showed up-regulation of a set of genes that are a part of the CESR-induced genes. The former regulation seems to be driven by nuclear tRNA surveillance since nuclear exosome inactivation by rrp6Δ dampens the up-regulation of the GAAC-related genes in the sla1Δ background. The authors stepped forward to isolate spontaneous suppressor mutants and found that a F32V mutation on the any1+ gene, encoding an arrestin-homologue, suppressed poor growth of the sla1Δ mutant on the NH4+ plate and supported growth of the surrounding any1+ cells. The any1-F32V sla1Δ cells incorporated Leu more efficiently even in the presence of NH4+ than the sal1Δ cells. The authors concluded that the tRNA processing factor La is involved in the GAAC response and Any1 specifically acts to support this signal transduction.
•Author response: We thank the Reviewer for careful reading of our paper and analysis of the data.

All the experiments seem to be performed technically rigorous and fit to the scientific standard of this field. Essentially, the data presented as figures and tables support the authors' notions. For example, they precisely described discrepancy between rrp6Δ effects on GAAC gene expression and those on growth in NH4+ media, which led to identification of a specific allele of any1+ as a suppressor of sla1Δ with a unique features. They appropriately cited previous paper especially in the transcriptome analyses of the sla1Δ cells. Thus, the reviewer essentially supports the publication of the manuscript in the journal of PLOS One. **Before publication, the following minor points should be amended:**
•Author response: We thank the Reviewer for noting support including appropriate citations of literature, as well as for the following noted points to be addressed which improve the clarity of the paper for the readership.

1) p. 23, line 581, p. 24, line 584; ug should be μg (micro gram).
•Author response: We thank the Reviewer for noticing our error. We fixed it.

2) p. 24, lines 583–584; it says that the Hybridization solution contained 100 μg/ml yeast RNA. Was the RNA prepared from budding yeast but not from fission yeast? Or, it might be "tRNA" but not "RNA."
•Author response: We thank the Reviewer. We clarified the text by noting that we used total RNA from Baker's yeast, obtained from Sigma. (I note here that it has been proven in my lab that it is indeed important to use RNA in the hybridization solution that is from a species that is phylogenetically distant from the source of the RNA attached to the membrane.)

3) p. 24, line 585; the company name should be "Fuji Film."
•Author response: We thank the Reviewer. We ammended the text accordingly.

4) The gene names and boxes in Tables 1–7 are color-coded. However, there is no explanation of the colors.
•Author response: To make the Tables uniform and standardized, we removed the color (it was used for organizational purposes during development but was superfluous and should have been removed prior to submission).

5) It is reader-friendly if the standard gene name of every gene listed in the lower section of Tables 2–7 is indicated. In addition, the reviewer is skeptical that the current style of the Tables 1–7 is appropriate for publication even on line. They should be adequately reshaped for publication.
•Author response: We appreciate this comment and the opportunity to reorganize the Tables which was helpful toward improving clarity of the paper. To address the second part of this comment we reshaped the tables as suggested, resulting in their reorganization and formalization to more succinct and slimmed down versions. I also note that seven tables have been designated as Supporting in the revised manuscript which we believe is appropriate

because they don't report primary data, but rather provide supportive information, such as gene names/descriptions and gene ontology secondary analysis for the multiple transcriptome categories analyzed in Fig. 1 (Supp Tables 1-7).

With regard to the first part of the comment, we added the PomBase designation for every gene listed in each of the five categories listed in Table 1 (120 genes) which covers all of the genes in the rest of the Tables and can thus serve as a source for handy cross-referencing; therefore, only the "Systematic ID" (e.g., SPAC57A10.10c) is listed in Tables 2-6, whereas Supp Table 7 also includes standard gene names because it includes a few genes not listed in Table 1. A footnote in Table 1 indicates that PomBase lists every gene as a Systematic ID and that when a gene name exists it is designated by three lower case letters followed by a number. In some cases, no gene name has yet been designated and only a product designation/description is provided https://www.pombase.org/

6) In Fig. 2B, it is difficult to recognize bar identity from pattern examples in its inset because the pattern examples were enlarged too much.
•Author response: We thank the Reviewer for pointing this out. We fixed it by revising the figure accordingly so that the patterns for each bar are clearly distinct.

7) In Fig. 6, the unit of the axis should be "pmol/10^7 cells."
•Author response: We thank the Reviewer for noticing our error. We fixed it.
