# Supplementary material for: The leucine-NH4+ uptake regulator Any1 limits growth as part of a general amino acid control response to loss of La protein by fission yeast
Source: PLoS One. 2021 Jun 21;16(6):e0253494. doi: 10.1371/journal.pone.0253494 (PMC8216550; doi:10.1371/journal.pone.0253494)
Supplement: Attachment — Submitted filename: Response to Reviewers PLoS One Any1R2.pdf [file pone.0253494.s010.pdf]

Cherkosova et al., Maraia, PONE-D-21-06328R1.
The leucine-NH4+ uptake regulator Any1 limits growth as part of a general amino acid stress response to loss of La protein by fission yeast

Point-by-point responses

I am sorry that my previous revision did not satisfy one point. On the other hand I am pleased to note that I believe that the several other revisions such as addition of quantification of multiple data points/sets, addition of *p*-values, R^2 values and SDs helped strengthen the paper, and I thank the reviewer and the review process for it.

Reviewer #1 noted "Cherkasova et al. have provided convincing response to issues raised in the previous round of review." The Reviewer noted that publication would be supported after one point would be addressed, and referred to our description of data on lines 437-440 discussing the quantifications in Fig 5. The short paragraph concluded with "If the authors cannot find statistical significance of aca1+ mRNA levels between lanes 15-16 and 17-18, they should reconsider the conclusion…. in lines 444-445."

Reponse: Consideration of this issue by myself and coauthors most knowledgeable on it led to our revision of the manuscript as suggested. The text that had been on lines 437-440 and conclusion on lines 444-445 were deleted. In addition, quantification numerals on lines 436-437 were also deleted. Additional edits to assimilate and accommodate the suggested considerations led to the revised manuscript in which the total extent of edits are limited to lines 431-474.
